# CONTRA-LATERAL PARADOXICAL PLEURAL EFFUSION DURING ANTITUBERCULOUS CHEMOTHERAPY

**DOI:** 10.4103/0970-2113.59593

**Published:** 2008

**Authors:** Vishal Chopra, Urvinderpal Singh, Dimple Chopra

**Affiliations:** Deptt. of Chest & Tuberculosis, Govt. Medical College, Patiala, Punjab, India

**Keywords:** Pleural effusion, Antituberculosis chemotheraphy

## Abstract

A 24-year old male developed left sided pleural effusion 10 days after the start of anti tubercular chemotherapy for right-sided pleural effusion and parenchymal lesion. This effusion seemed to be a paradoxical response as it resolved on follow up.

## INTRODUCTION

Paradoxical response is referred to an unusual expansion or formation of a new lesion during successful anti-tubercular chemotherapy^1^. This response has been described in cases of tubercular lymph-adenopathy^2^ and intra-cranial tuberculoma^3^ though it has been very rarely reported in cases of pleural effusion^4^–^7^. The pleural effusion has been documented to occur 3-4 weeks after the start of ATT^6^,^7^ although it developed within 10 days of initiating anti tuberculosis treatment in our case. To our knowledge about 15 cases have been reported so far in literature hence this case report.

## CASE REPORT

**Fig. 1:**
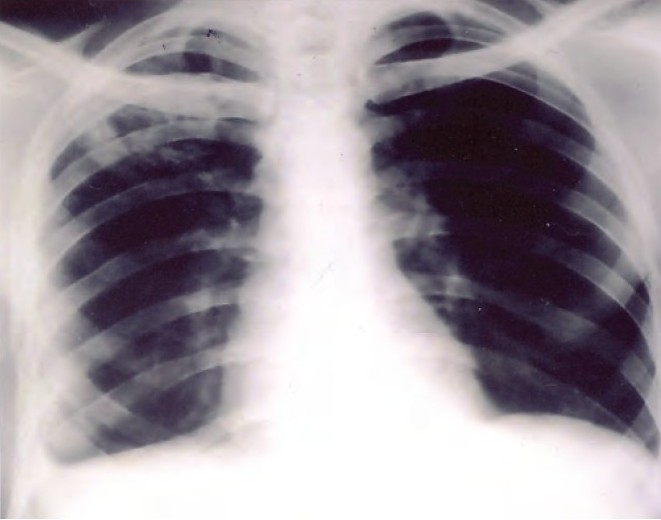
Xray chest PA view showing heterogeneous opacity in the right upper zone with blunting of the right costophrenic angle.

**Fig. 2:**
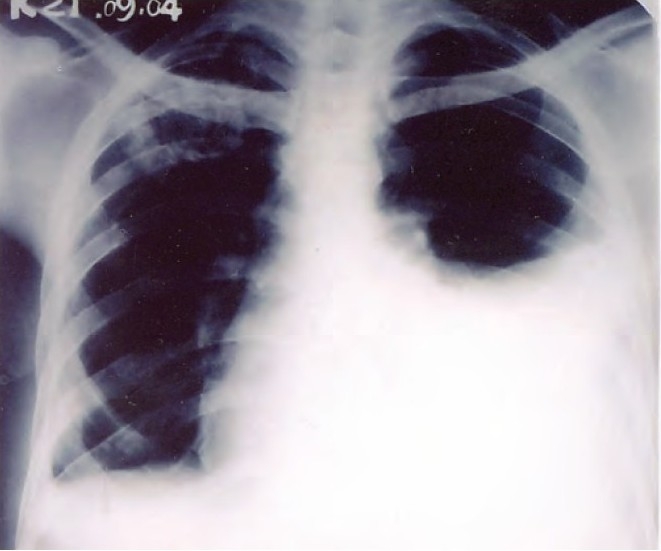
Xray chest PA view showing heterogeneous opacity in the right upper zone with blunting of the right costophrenic angle and moderate pleural effusion on the left side.

**Fig. 3:**
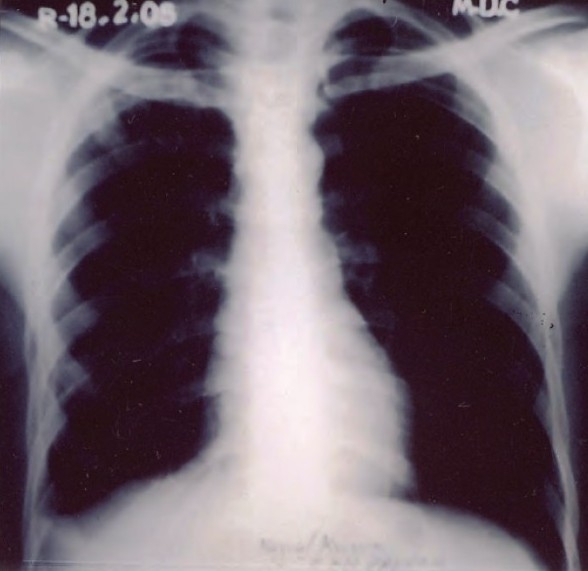
Xray chest PA view at 4 months showed clearing of the pleural on the left side with blunting of right costo-phrenic angle due to thickening of the pleura.

A 24-year old male patient presented with complaints of cough with expectoration for one month, fever for 20 days and pain on the right side of the chest of five days duration. General physical examination revealed no abnormality. Examination of the chest revealed crepitations in the right infra-clavicular area and signs of right-sided pleural effusion. Chest radiograph showed a heterogeneous opacity in the right upper zones with blunting of the right costophrenic angle (fig. 1). Sputum examination was positive for acid-fast bacilli. Pleural fluid revealed straw colored exudative effusion with a predominance of lymphocytes. Patient was put on Category 1 treatment for tuberculosis as per Revised National Tuberculosis Control Programme (RNTCP) guidelines. After eight days of start of therapy, the patient presented with sudden onset of pain on the left side of chest and dyspnoea. Chest radiograph revealed a pleural effusion on the left side (fig. 2). A straw colored effusion was aspirated which was exudative in nature with predominance of lymphocytes. The patient was reassured and ATT was continued as before. After 12 weeks of ATT there was significant clinical and radiological improvement bilaterally. A chest radiograph at 4 months showed clearing of the pleural effusion on the left side with blunting of right costo-phrenic angle probably due to thickening of the pleura (fig. 3).

## DISCUSSION

Paradoxical response is referred to an unusual expansion or formation of a new lesion during successful anti-tubercular chemotherapy^1^. This response has been described in cases of tubercular lymphadenopathy but has been rarely reported in cases of pleural effusion. The paradoxical increase of the disease is documented to occur weeks or months following the start of ATT^1^–^3^.

Previous reporters established an incidence of 16% in cases of tubercular pleural effusion^4^ that is far less than 30% reported for tubercular lymphadenopathy^3^. About 15 cases have been reported so far in literature and in all the reported cases the paradoxical effusion occurred in the same hemithorax but in our case the response was on the opposite side.

Rupture of subpleural abcesses into the pleural space along with hematogenous dissemination and rupture of caseous lymph nodes into the pleural space has been proposed as an explanation^8^. Speculations have lead to the interaction between host's immune response and direct effects of mycobacterial products as an explanation to this response^9^ by some workers. Other mechanism put forward is the ‘immunological rebound’ by which improved CMI after treatment coincides with an excessive antigen load (bacterial cell wall residues) resulting from rapid bacterial lysis^1^,^4^,^10^. It has also been suspected that bactericidal drugs like Isoniazid & Rifampicin could be worse offenders than bacteriostatic drugs. INH is well known to induce lupus though the reported cases are rare. An elevated level of ANA and a decreased level of CH50 were found in the effusion fluid which are characteristic of lupus pleuritis^5^.

A more detailed study is warranted to understand the etiopathogenesis of paradoxical worsening in cases of tuberculosis. The present case highlights the importance of understanding and knowing the pathological evidence of this clinical process during the management of pulmonary tuberculosis.

Thus it is concluded that the development of contralateral pleural effusion during the treatment for tuberculous effusion is very rare and no change in the treatment is required until some other disease is suspected.
